# Systemic Immune Effects of Titanium Dioxide Nanoparticles after Repeated Intratracheal Instillation in Rat

**DOI:** 10.3390/ijms15046961

**Published:** 2014-04-22

**Authors:** Yanyun Fu, Yanqiu Zhang, Xuhong Chang, Yingjian Zhang, Shumei Ma, Jing Sui, Lihong Yin, Yuepu Pu, Geyu Liang

**Affiliations:** 1 Key Laboratory of Environmental Medicine Engineering, Ministry of Education, School of Public Health, Southeast University, Nanjing 210009, Jiangsu, China; E-Mails: fckxxz@gmail.com (Y.F.); zyqtxl1986@gmail.com (Ya.Z.); zhangyingjian321@gmail.com (Yi.Z.); m837862513@gmail.com (S.M.); sj331622547@gmail.com (J.S.); lhyin@seu.edu.cn (L.Y.); yppu@seu.edu.cn (Y.P.); 2 Department of Epidemiology and Health Statistics, School of Public Health, Southeast University, Nanjing 210009, Jiangsu, China; E-Mail: cxhpublic@gmail.com

**Keywords:** nano-TiO_2_, systemic immune response, lymphocyte proliferation, cytokines

## Abstract

The potential immune effects of titanium dioxide nanoparticles (nano-TiO_2_) are raising concern. Our previous study verified that nano-TiO_2_ induce local immune response in lung tissue followed by intratracheal instillation administration. In this study, we aim to evaluate the systemic immune effects of nano-TiO_2_. Sprague Dawley rats were treated by intratracheal instillation with nano-TiO_2_ at doses of 0.5, 4, and 32 mg/kg body weight, micro-TiO_2_ with 32 mg/kg body weight and 0.9% NaCl, respectively. The exposure was conducted twice a week, for four consecutive weeks. Histopathological immune organs from exposed animals showed slight congestion in spleen, generally brown particulate deposition in cervical and axillary lymph node. Furthermore, immune function response was characterized by increased proliferation of T cells and B cells following mitogen stimulation and enhanced natural killer (NK) cell killing activity in spleen, accompanying by increased number of B cells in blood. No significant changes of Th1-type cytokines (IL-2 and INF-γ) and Th2-type cytokines (TNF-α and IL-6) were observed. Intratracheal exposure to nano-TiO_2_ may be one of triggers to be responsible for the systemic immune response. Further study is needed to confirm long-lasting lymphocyte responses and the potential mechanisms.

## 1. Introduction

Nanomaterials are engineered structures with at least one dimension of 100 nanometers or less [1]. With the rapid development of the nanotechnology industry, more and more nanoparticles have generally come into our life through their different approaches. At the same time, safety in nanoparticles is attracting more and more attention.

Because of its unusual physicochemical properties, titanium dioxide nanoparticles (nano-TiO_2_) have a wide range of applications in many fields, including cosmetics, pharmaceutical, and paint industries. Previous *in vivo* studies have shown that nano-TiO_2_ particles can easily travel throughout the body, penetrate cell membranes, lodge in mitochondria, deposit in target organs, and may cause different degrees of organ damage, including lung, liver, kidney and brain [2–10]. Some *in vitro* studies also have shown that nano-TiO_2_ can cause oxidative stress, DNA damage and enzymatic activity changes, followed by cell apoptosis or necrosis [11–13]. Recently, it has been reported that exposure to nano-TiO_2_ in experimental animals can damage pulmonary macrophages, induce splenocyte apoptosis, promote reactive oxygen species (ROS) accumulation, change cytokine production and decrease immune function [14–18]. However, the researches on immunotoxicity of nano-TiO_2_ are still rarely reported.

Our previous study evaluated the local immune function of rat exposed to nano-TiO_2_ by intratracheal instillation [19]. The phagocytic ability of the pulmonary alveolar macrophages was increased when they were exposed to low-dose of nano-TiO_2_ and decreased when they were exposed to high dose of nano-TiO_2_. In addition, nano-TiO_2_ damaged the cellular structure, reduced chemotactic ability, decreased expression of both Fc receptors and major histocompatibility complex (MHC)-class II molecules and increased nitric oxide (NO) and tumor necrosis factor alpha (TNF-α) secretion of macrophages, which suggest that nano-TiO_2_ disrupt the functions of macrophages associated with pulmonary non-specific and specific immunity. On that basis, this study was conducted to assess systemic immune response to repeated pulmonary nano-TiO_2_ exposure in rats.

## 2. Results

### 2.1. Histopathological Examination

**Figure 1.**
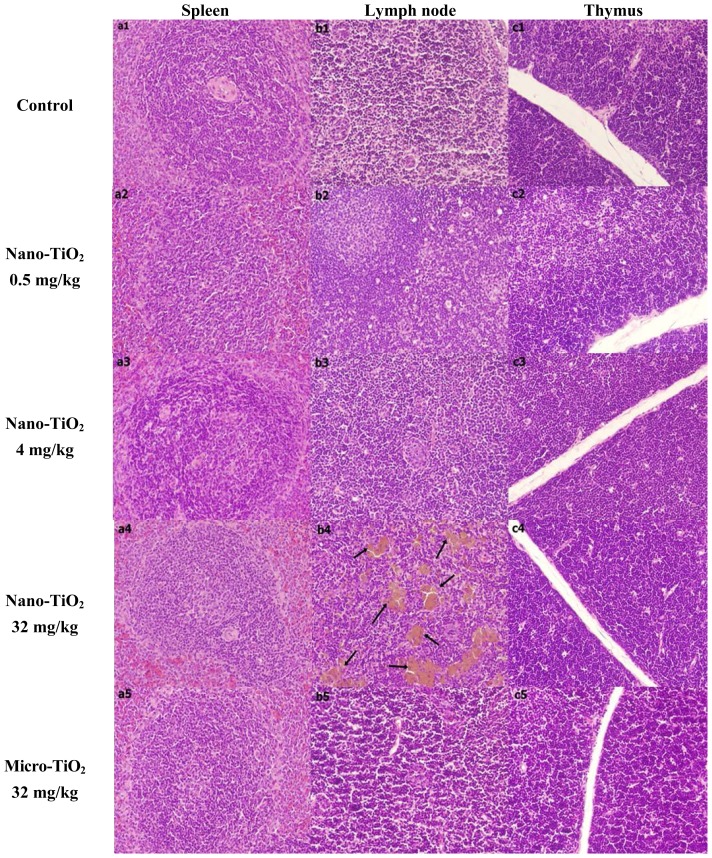
Histopathology of the tissue in Sprague-Dawley (SD) rat caused by an intratracheal instillation with nano-TiO2 for 28 days (200×). Hematoxylin and eosin stains of spleen (**a1**–**a5**); lymph node (**b1**–**b5**) and thymus (**c1**–**c5**) tissues of rat. Black arrows point to brown particulate deposition.

Histopathological evaluation was performed in various immune organs including spleen, cervical and axillary lymph node and thymus (Figure 1). Rats from high-dose nano-TiO_2_ exposure showed slight congestion of the splenic sinus. In cervical and axillary lymph node, the brown particulate was generally aggregated in different sizes in rat exposed to 32 mg/kg nano-TiO_2_. No histopathological changes were found in thymus in all groups.

### 2.2. Mitogen Stimulated Lymphocyte Proliferation Assay

**Figure 2.**
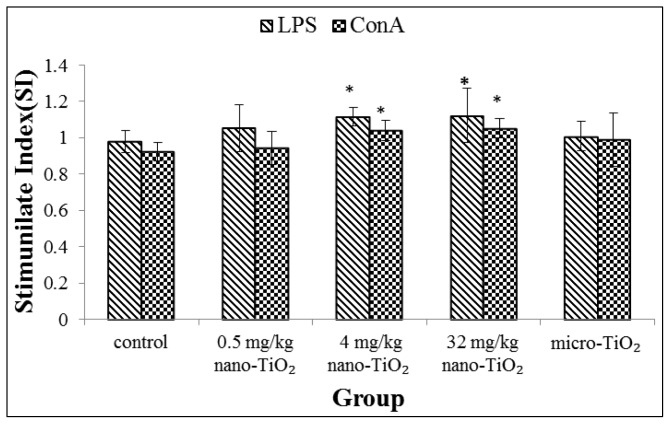
Lymphocyte proliferation in the spleen from rats exposed to nano-TiO_2_ for 28 days by intratracheal instillation. ^*^ represents significantly different from control group (*p* < 0.05).

In this assay, lipopolysaccharide (LPS) and concanavalin A (ConA) are usually used to analyze the proliferation of B- and T-lymphocyte, respectively. The effects of treatment with nano-TiO_2_ particles on spleen-derived lymphocyte proliferation in the rat are shown in Figure 2. As the exposure dose of nano-TiO_2_ was increased, the B- and T-lymphocyte proliferation stimulated by mitogen was increased. Significant difference in the B- and T-lymphocyte proliferation ability was found in the groups exposed to 4 and 32 mg/kg nano-TiO_2_ compared with the control group (*p* < 0.05).

### 2.3. Natural Killer (NK) Cell Activity Assay

**Figure 3.**
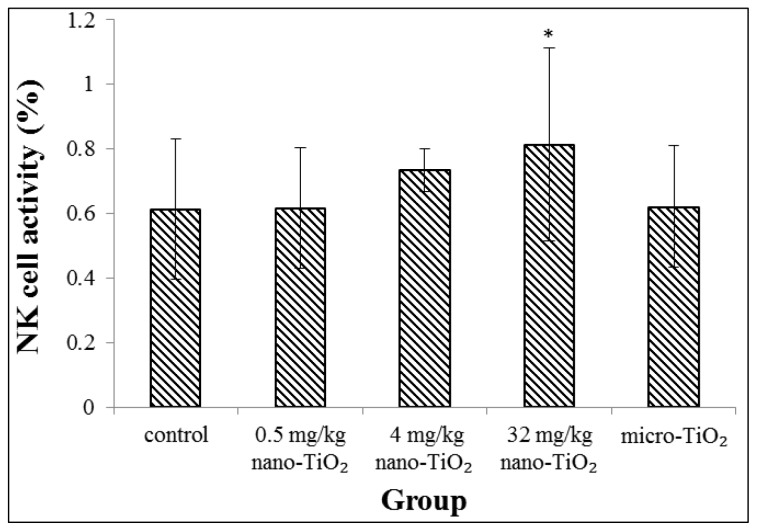
Natural killer (NK) cell activity in the spleen from rats exposed to nano-TiO_2_ for 28 days by intratracheal instillation. ^*^ represents significantly different from control group (*p* < 0.05).

The effects of treatment with nano-TiO_2_ particles on spleen-derived natural killer (NK) cell activity in the rat are shown in Figure 3. The NK cell activity was increased with the rise of exposure dose of nano-TiO_2_ particles. Significant difference in the NK cell activity was found in the groups exposed to 32 mg/kg nano-TiO_2_ compared with the control group (*p* < 0.05).

### 2.4. Lymphocyte Population of Peripheral Blood

**Figure 4.**
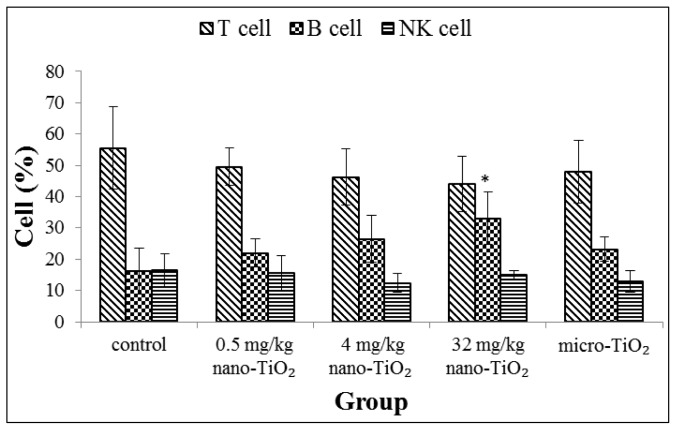
Lymphocyte population distribution of peripheral blood from rats exposed to nano-TiO_2_ for 28 days by intratracheal instillation. ^*^ represents significantly different from control group (*p* < 0.05).

The results of lymphocyte population distribution in the peripheral blood of rat treated with nano-TiO_2_ are shown in Figure 4. The B-lymphocyte population distribution was increased with the rise of exposure dose of nano-TiO_2_. A significant increase of B-lymphocyte distribution was found in the groups exposed to 32 mg/kg nano-TiO_2_ compared with the control group (*p* < 0.05). No significant differences in distribution of T-lymphocyte populations and NK cell populations were found between exposure groups and control group (*p* > 0.05).

### 2.5. Cytokine Production in Blood

**Figure 5.**
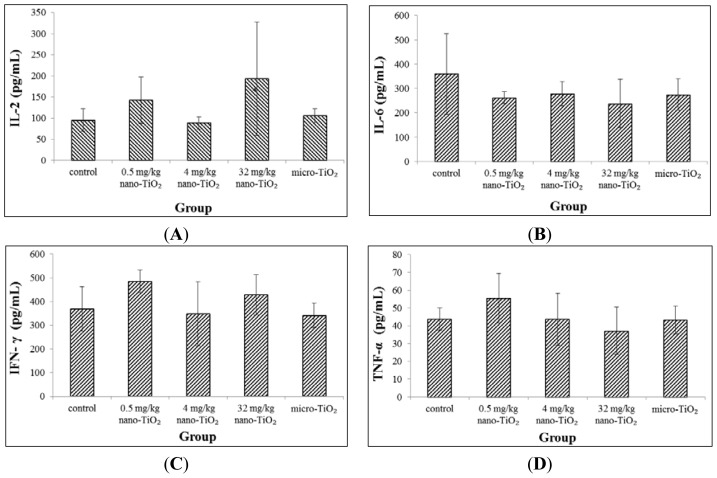
Cytokines expression in the peripheral blood from rats exposed to nano-TiO_2_ for 28 days by intratracheal instillation. (**A**) the changes of IL-2 expression; (**B**) the changes of IL-6 expression; (**C**) the changes of IFN-γ expression; and (**D**) the changes of TNF-α expression.

The effects of nano-TiO_2_ on the production of cytokines in blood were evaluated. No significant changes of IL-2, IL-6, IFN- γ and TNF-α expression were observed (Figure 5).

## 3. Discussion

Increased use of artificial nanoparticles in a wide range has introduced a potential inhaled pollutant. Although the reports on toxicity of nanoparticles are now increasing, immunity-related responses of nanoparticles have not been well studied. In this study, we focused on the systemic immune response induced by repeated exposure of titanium dioxide nanoparticles, involved in immune organ formation, lymphocyte proliferation, lymphocyte distribution and cytokine induction.

It has been known that the toxicological effects of nanoparticles are closely related to their specific physicochemical properties. Nano-TiO_2_ seemed to be agglomerated in solution state, which might influence evaluation of biological response of nano-TiO_2_. In our study, we found that 0.9% NaCl buffer is a suitable solution for preparation of nano-TiO_2_ dispersions and subsequent biological investigation because the solution buffer with 0.9% NaCl maintained a better dispersion. Nano-TiO_2_ suspension solution used for the intratracheal instillation in this study showed less aggregation. The mean diameter of the agglomerated nano-TiO_2_ was about 200 nm, which was not micrometer level, but nano level.

The immune organs play a vital role during the body defense progress for xenobiotic. Spleen is the largest lymphoid organ and important in both innate and adaptive immune. A few evidences have been reported that nano-TiO_2_ could produce spleen response. Li *et al.* reported that nano-TiO_2_ exposure at doses of 50 and 150 mg/kg by intraperitoneal injection for consecutive 45 days induced obvious congestion and lymph nodule proliferation in the mouse spleen [18]. Sang *et al.* showed that doses of 2.5, 5 and 10 mg/kg nano-TiO_2_ administrated by intragastric injection for 90 days produced congestion, white pulp rarely, disperative replication of white pulp and anemia of red pulp in the mouse spleen [17]. Chen *et al.* found that a large number of TiO_2_ particles accumulated in spleen, and caused a mass of neutrophilic cells in spleen tissues and a severe spleen lesion after nano-TiO_2_ exposure with higher doses (324–2592 mg/kg) by an intraperitoneal injection for 7 days [8]. Wang *et al.* observed that 14 days after a large dose of 5 g/kg of nano-TiO_2_ was administrated by a single intragastric injection, resulted in splenic accumulation of nano-TiO_2_, but did not cause abnormal pathology changes in the mouse spleen [7]. The histopathological results of this study showed that the repeated intratracheal instillation administration of various doses of nano-TiO_2_ can induce slight congestion of spleen, consistent with above studies. We did not observe the severe injury of spleen, which may be due to the exposure dose or time of nano-TiO_2_ (no more than 32 mg/kg, 28 days) in our study which was lower than that in other studies. In addition, we also observed that nano-TiO_2_ may cause significant accumulation in the rat lymph node, while there are no abnormal pathology changes in the micro-TiO_2_ exposure group. Van Ravenzwaay *et al.* also reported nano-TiO_2_ (14% rutile, 86% anatase) accumulation in lymphoid tissue after inhalation exposure in the rat [20]. These results indicated that inhaled nano-TiO_2_ could translocate throughout the body quickly and tend to accumulate in immune organ, which possibly through uptake by migratory antigen presenting cells, because many toxicological studies have observed that macrophages or foreign-body giant cells appeared in lung tissue as exposure doses increased [21,22].

We have previously reported altered local immune cell function following intratracheal instillation of nano-TiO_2_. It was observed that nano-TiO_2_ were uptake in pulmonary macrocrophages, consistent with pulmonary macrophages as the first-line defense against inhalation of particles. Function detection showed that exposures to nano-TiO_2_ increased the phagocytic ability of the pulmonary macrophages [19]. In this study, immune function measurements on spleen-derived cells showed increased proliferation of T cells and B cells following mitogen stimulation and enhanced NK cell killing activity, which was accompanied by increased B cells number in blood. Lymphocyte proliferation is an important phase in the immune response. The results indicated that nano-TiO_2_ could trigger systemic immune responses. Recent studies have revealed that exposure to nanoparticles can stimulate immune cell. Lee *et al.* reported that mesoporous silica nanoparticles led to significant splenocyte proliferation to the lymphocyte mitogens when administered by intraperitoneal injection in female BALB/c mice for 4 weeks [23]. Park *et al.* showed an increase in numbers of B cells in splenocytes and in blood after mice were treated with nano-TiO_2_ by a single intratracheal instillation [24]. Wang *et al.* researched that increases T cells in the spleen of C57BL/6 mice dependent on nanomaterial comparison of graphene and carbon nanotubes [25]. Gustafsson *et al.* discussed an increase in numbers of NK cells in lung after exposure to nano-TiO_2_ by intratracheal installation [26]. However, Sang *et al.* noted lymphocytes (including T, B lymphocyte and NK cell) of whole blood in mice were significantly decreased following nano-TiO_2_ exposure for 90 consecutive days by intragastric administrations [17]. Differences in response may be attributed to route of administration, dose and exposure time.

Cytokines are very important regulators of the immune responses and serve to maintain balance of immune system. After adaptive immune system was triggered, the activated T cells were differentiated into helper T cells and cytotoxic T cells. Then, helper T cells were differentiated into Th1 cell and Th2 cell. Th1 and Th2 cell trigger cellular and humoral immunity by secret cytokines, respectively. It was reported that IL-2 and IFN-γ levels in serum levels was significantly increased in rat treated with nano-TiO_2_, indicating that nano-TiO_2_ exposure may trigger Th1 immune response [26]. However, another recently report showed that nano-TiO_2_ induced a Th2 cell response in mice [27]. In this study, we analyzed the Th1-type cytokines (IL-2 and INF-γ) and Th2-type cytokines (TNF-α and IL-6). However, no significant changes of these cytokines expression were observed throughout the experimental period. The discrepancy is likely explained by species differences, nano-TiO_2_ particle differences, especially observation time differences. Gustafsson *et al.* reported that most cytokine expression at days 1–2 and a second response at day 16 of TNF-α after nano-TiO_2_ exposure [26]. Park *et al.* also noted IL-2 and IL-4 were increased in a time-dependent manner [24]. In the future, dynamic investigations will be performed to understand the mechanism between Th-related cytokines and systemic immune response with nano-TiO_2_ exposure.

## 4. Experimental Section

### 4.1. Preparation of Nano-TiO_2_

**Figure 6.**
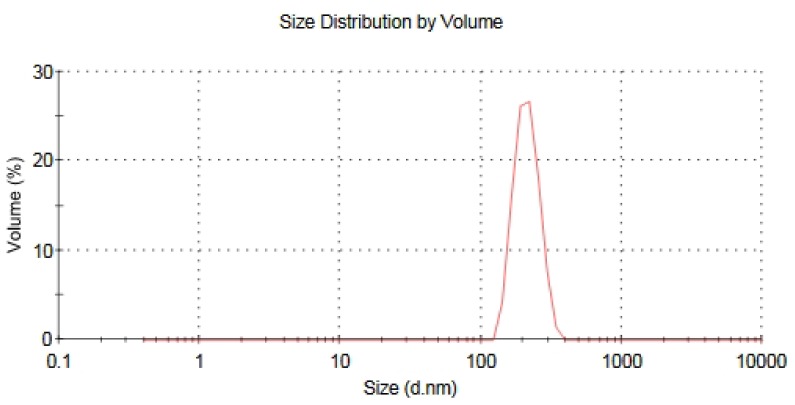
Particle size distribution of nano-TiO_2_ in NaCl suspension.

Manufactured nano-TiO_2_ (P25) was purchased from Degussa Corporation (Hanau, Germany). Micro-TiO_2_ of size 1–2 μm was purchased from Beijing DK nano technology Co., Ltd. (Beijing, China). According to the information provided by the supplier, nano-TiO_2_ is a fine white powder with mean diameter of 21 nm (Table 1). The characteristics of the nano-TiO_2_ were found in our previously study [28]. Nano-TiO_2_ was heated to 121 °C for 20 min to reduce the risk of bacterial contamination. TiO_2_ powder was dispersed into an aqueous solution buffered with 0.9% (*w*/*v*) sodium chloride (NaCl) solution. For sufficiently disperse particles, solutions containing TiO_2_ particles were sonicated for 5 min at 30% amplitude by Sonicator ultrasonic processor (Model S-4000, Misonix, Inc., Farmingdale, NY, USA). Size distribution of nano-TiO_2_ suspension was also analyzed using Malvern Instruments Zetasizer Nano ZS90 (Malvern Instruments Ltd., Worcestershire, UK). The mean diameter of nano-TiO_2_ suspended in 0.9% NaCl was about 200 nm (Figure 6).

### 4.2. Animals and Treatment

Male Sprague-Dawley (SD) rats, with an average body weight of 200–220 g, were provided from Liaoning Chang Sheng Biotechnology Co., Ltd. (Benxi, China). The animal room was maintained at 20 ± 2 °C, 60% ± 10% relative humidity and a 12 h light/dark cycle. Rats were acclimatized for 5 days before experimentation. Procedures complied with the national regulations related to animal welfare. Forty rats were randomly divided into five groups. The control group was treated with 0.9% *w*/*w* NaCl solution. The experimental groups were treated with 0.5, 4, 32 mg/kg nano-TiO_2_ and 32 mg/kg micro-TiO_2_, respectively. All groups were performed to repeated exposure (twice a week, for four consecutive weeks) by intratracheal instillation. The volume of intratracheal instillation was 0.1 mL/100 g. Four weeks later, the rats were sacrificed by exsanguination via the abdominal aorta after being anesthetized by ether. Spleen, cervical and axillary lymph node, thymus, and whole blood were collected for further assay.

### 4.3. Histopathological Examination

All histopathological tests were performed using standard lab-oratory procedures. The spleen, lymph node and thymus tissues were fixed in a 10% formalin solution for one week. Then, the tissues were embedded in paraffin blocks, and then sectioned into 5 μm slices and mounted on glass slides. After hematoxylin–eosin (HE) staining, the slides were examined using an optical microscope (Olympus, Tokyo, Japan). The identity and analysis of the pathology slides were blind to the pathologist.

### 4.4. Preparation of Spleen-Derived Lymphocyte Populations

Single-cell lymphocyte populations were prepared from the spleen of rat. Spleen was dissociated on a steel mesh in Hanks’ balanced salt solution (Beyotime, Nantong, China). The splenocyte that passed through the mesh were centrifuged at 2000 rpm for 5 min. The supernatant was discarded and cells were resuspended in the Hanks’ medium. The red blood cell lysis buffer (Beyotime, Nantong, China) was added to lyse the erythrocytes. Then, the cell suspension was filtered and washed to remove cell debris. Finally, cells were resuspended in RPMI1640 [with 10% fetal bovine serum (FBS)] (Hyclone, Victoria, Australia). Cell viability was assessed using the trypan blue exclusion test and routinely found to contain <5% dead cells. The cell density was adjusted to 5 × 10^6^ cells/mL for further assay.

### 4.5. Mitogen Stimulated Lymphocyte Proliferation Assay

Lymphocyte proliferation responses to mitogens (lipopolysaccharide, LPS and concanavalin A, ConA) were evaluated with the CCK-8 assay. For each spleen sample, lymphocyte were placed into a U-bottom 96-well plate in triplicate at a density of 1 × 10^6^ cells/mL with a total volume of 90 μL. 10 μL LPS (L2630, Sigma, St. Louis, MO, USA) or ConA (C2010, Sigma, St. Louis, MO, USA) at a concentration of 20 μg/mL was added to wells, respectively. Supplemented RPMI1640 media were added to control wells. Plates were briefly mixed on a plate shaker and then placed in a 5% CO_2_, 37 °C incubator for 72 h. Then, CCK-8 was added and incubated for an additional 4 h. Following incubation, spectrophotometric data were measured using an automatic microplate reader (type MRX, Dynex Technologies Company, Chantilly, VA, USA) at a wavelength of 450 nm.

### 4.6. Natural Killer (NK) Cell Activity Assay

NK cell activity was evaluated by lactate dehydrogenase (LDH) release assay. Human erythroleukemia cell line K-562 (Chinese Academy of Sciences, Shanghai, China) was used as a target cell in this assay because it is sensitive to the cytotoxicity of NK cells. K562 cells were routinely cultured in RPMI1640 medium (with 10% FBS) (Hyclone, Victoria, Australia) and were plated at a concentration of 1 × 10^5^ cells/mL in U-bottom 96-well plates (Corning, Shanghai, China). Splenocytes were plated at 5 × 10^6^ cells/mL for an effector to target ratio (E:T) of 50:1. Two controls were placed for the assay, including a spontaneous control (target cells + RPMI1640 medium) and a maximum spontaneous control (target cells + 1% NP40 lysis buffer). Plates were incubated at 5% CO_2_, 37 °C for 20 h and then centrifuged at 2000 rpm for 5 min. One hundred μL the supernatant was removed to the new plate, followed by 100 μL LDH matrix liquid and 30 μL 1 M HCL was added to each well. Finally, optical density (*OD*) was measured using an automatic microplate reader at a wavelength of 490 nm. Percentage of cytotoxicity was calculated by:

(1) Cytotoxicity %=(sample OD-spontaneous OD)/(maximum OD-spontaneous OD)×100%

### 4.7. Lymphocyte Population Analyses in Blood

Lymphocyte population distribution in peripheral blood was examined using flow cytometry method. All monoclonal antibodies were purchased from eBioscience (San Diego, CA, USA). T cells (CD3, eBioG4.18), B cells (CD45RA, OX33), NK cells (CD161, clone 10/78) were identified using directly conjugated anti-rat antibody [29]. Erythrocytes in the blood were lysed with red blood cell lysis buffer. Then, cells were washed with PBS buffer. Finally, each sample was fixed with 0.1% paraformaldehyde and analyzed by flow cytometry (BD Biosciences, Franklin Lakes, NJ, USA).

### 4.8. Cytokines Analyses in Blood

Serum samples were obtained from peripheral blood by centrifugation at 3000 rpm for 10 min at 4 °C and immediately frozen at −80 °C for further detection. IL-2, IL-6, IFN-γ and TNF-α were measured by a MILLIPLEX MAP Rat Cytokine Kit (Millipore, Billerica, MA, USA) according to the manufacturers’ instructions.

### 4.9. Statistical Analysis

Statistical analysis was done by SPSS16.0 software (SPSS Inc., Chicago, IL, USA). The differences of means among groups were analyzed by One-way analysis of variance method (ANOVA). The comparisons between experimental group and control group were made by LSD test. The results were regarded as statistical significant as *p* < 0.05.

## 5. Conclusions

In summary, nano-TiO2 induced pathological changes of spleen and lymph node, including slight congestion and brown particulate accumulation. Furthermore, increased proliferation of spleen-derived T cells and B cells following mitogen stimulation and enhanced NK cell killing activity were observed by repeated instillation of nano-TiO_2_. Number of B cell was also increased in the blood. The result suggested that nano-TiO_2_ may be one of triggers to be responsible for the systemic immune response. The study served to improve the overall understanding of the immune response associated with inhalable nano-TiO_2_ and provided new strategy for risk assessment of nano-TiO_2_. More work needs to be done to evaluate the potential mechanism for this response.

## Figures and Tables

**Table 1.** Characterization of TiO_2_ particles.

Particle	Nano-TiO_2_	Micro-TiO_2_
supplier	Degussa	DK nano
size (nm)	21 nm	1–2 μm
crystalline form	80% anatase/20% rutile	anatase
specific surface area (m^2^/g)	50	18
purity (%)	>99.5	>99.9
*Z*-Average (d.nm)	195.2	–
PDI *	0.066	–

* The polydispersity index (PDI) describes the width of the particle size distribution.
